# Regulation of the microvascular circulation in the leg muscles, pancreas and small intestine in rats

**DOI:** 10.1186/s40064-015-1102-8

**Published:** 2015-06-26

**Authors:** Hisashi Maeda, Tomoyuki Kurose, Seiichi Kawamata

**Affiliations:** Graduate School of Biomedical and Health Sciences, Hiroshima University, Kasumi 1-2-3, Minami-ku, Hiroshima, 734-8551 Japan; Department of Anatomy and Histology, Institute of Biomedical and Health Sciences, Hiroshima University, Kasumi 1-2-3, Minami-ku, Hiroshima, 734-8551 Japan

**Keywords:** Capillary, Blood flow, *Lycopersicon esculentum* lectin, PECAM-1 (platelet endothelial cell adhesion molecule-1), CD31, Immunohistochemistry

## Abstract

To study the microvascular circulation, we examined the proportion of open and functioning capillaries in the leg muscles, pancreas and small intestine of anesthetized rats. Fluorescein isothiocyanate (FITC)-labeled *Lycopersicon esculentum* lectin was injected into the heart and allowed to circulate for 3 min to label open and functioning capillaries. Specimens were removed, frozen, sectioned and double-immunostained. Using one section, open and functioning capillaries were detected by immunostaining for this lectin bound to endothelial cells, while all capillaries were visualized by immunostaining for platelet endothelial cell adhesion molecule-1 (PECAM-1 or CD31). These capillaries were semi-automatically detected and counted by fluorescence microscopy. The percentages of open and functioning capillaries were as follows: the soleus muscle, 93.0 ± 5.5%; superficial zone of the gastrocnemius muscle, 90.8 ± 6.2%; deep zone of the gastrocnemius muscle, 95.6 ± 4.0%; the plantaris muscle, 94.1 ± 2.7%; the pancreas, 86.3 ± 11.7%; and the small intestine, 91.1 ± 4.9% (n = 8, each). There was no significant difference among these data by the Kruskal–Wallis test. This study clearly demonstrated that the proportions of open and functioning capillaries are high and similar among the leg muscles, pancreas and small intestine in spite of their structural and functional differences. This finding agrees with previous studies and supports the notion that the microvascular circulation is mainly controlled by changing of the blood flow in each capillary rather than changing the proportion of open and functioning capillaries.

## Background

The blood supply is vitally important for the transport of essential materials and metabolic waste. Capillaries are densely distributed throughout most tissues and organs. Adapting to physiological conditions, the microvascular circulation is elaborately regulated. For example, blood flow of skeletal muscles is low at rest, but drastically increases upon exercise (Nilsson and Ingvar 1967; Terjung and Engbretson 1988; Musch and Terrell 1992; Hawker and Egginton 1999; Kindig et al. 2002; Richardson et al. 2003) and local warming (Edholm et al. 1956; Minson et al. 2001; Fiscus et al. 2005). Furthermore, blood flow in various tissues and organs is significantly different. The blood flow of rat per unit time and weight is reported as follows (Musch and Terrell 1992): the soleus muscle, 140 ± 18; superficial zone of the gastrocnemius muscle (gastrocnemius, white), 10 ± 2; deep zone of the gastrocnemius muscle (gastrocnemius, red), 60 ± 12; the plantaris muscle, 15 ± 3; the pancreas, 148 ± 22; and the small intestine, 247 ± 29 ml min^−1^ 100 g^−1^ (mean ± SE). However, the regulation mechanism of blood flow in the capillary bed is not fully understood.

The blood flow considerably changes depending on the blood flow velocity (Honig et al. 1980; Richardson et al. 2003) and the size of blood vessels (Kano et al. 2005). These factors are influenced by the blood pressure, nerve activity (Minson et al. 2001; Johnson et al. 2005), vasoactive substances (Minson et al. 2001; Koganezawa et al. 2006), blood viscosity, contraction of the skeletal muscle (Nilsson and Ingvar 1967), tissue temperature (Karunakara et al. 1999; Koganezawa et al. 2006; Keller et al. 2010) and so on. The proportion of open and functioning capillaries, i.e., the percentage of capillaries supporting continuous red blood cell flow (Kindig et al. 2002), is also assumed to influence blood flow. If non-flowing capillaries in resting muscle are recruited during muscle contraction, the proportion of open and functioning capillaries will increase (Honig et al. 1980), resulting in an increase of blood flow. On the other hand, Poole et al. (2013) stated that the majority of capillaries support blood flow at rest and are not available to be recruited. The proportion of open and functioning capillaries has been repeatedly measured in skeletal muscles (Renkin et al. 1981; Kayar and Banchero 1985; Kindig et al. 2002; Kano et al. 2005; Maeda et al. 2014) and remains controversial (Poole 2014; Barrett et al. 2014).

Concerning microvascular studies, *Lycopersicon esculentum* lectin is well known to bind endothelial cells and has been employed in combination with PECAM-1 immunostaining (Hashizume et al. 2000; Morikawa et al. 2002; Inai et al. 2004; Mazzetti et al. 2004; Nakamura-Ishizu et al. 2008; Maeda et al. 2014; Robertson et al. 2015). In this method, the plasma flow results in lectin binding to endothelial cells, even without moving red blood cells. However, Kawaguchi et al. (2012) observed similar spatiotemporal behaviors for the plasma and red blood cells, being labeled by water-soluble Qdot and fluorescein isothiocyanate (FITC), respectively. Poole et al. (2013) found that the majority of capillaries support blood flow in resting muscle, although plasma and red blood cells travel at different speeds through capillaries. Thus, it is plausible that plasma and red blood cells flow through open and functioning capillaries in most capillaries.

In our previous study, two serial cross sections were used to measure the proportion of open and functioning capillaries in rat leg muscles (Maeda et al. 2014). In paired cross sections of skeletal muscles, the number, location and direction of blood vessels are almost the same since most blood vessels run along the muscle fiber’s long axis. In the majority of tissues and organs, however, blood vessels run in various directions and the profile of the capillary bed considerably differs between two adjacent sections. Then, we employed a double-immunostaining method that uses only one section instead of using two adjacent sections. This method can be used for not only skeletal muscles but also organs containing randomly oriented capillaries. In the present study, we measured the proportion of open and functioning capillaries in leg muscles as a muscle tissue, the pancreas as an example of a parenchymal organ and the small intestine as an example of a luminal organ, and examined whether or not these data differ among these tissues and organs, reflecting their structural and functional differences.

## Results

**Figure 1:**
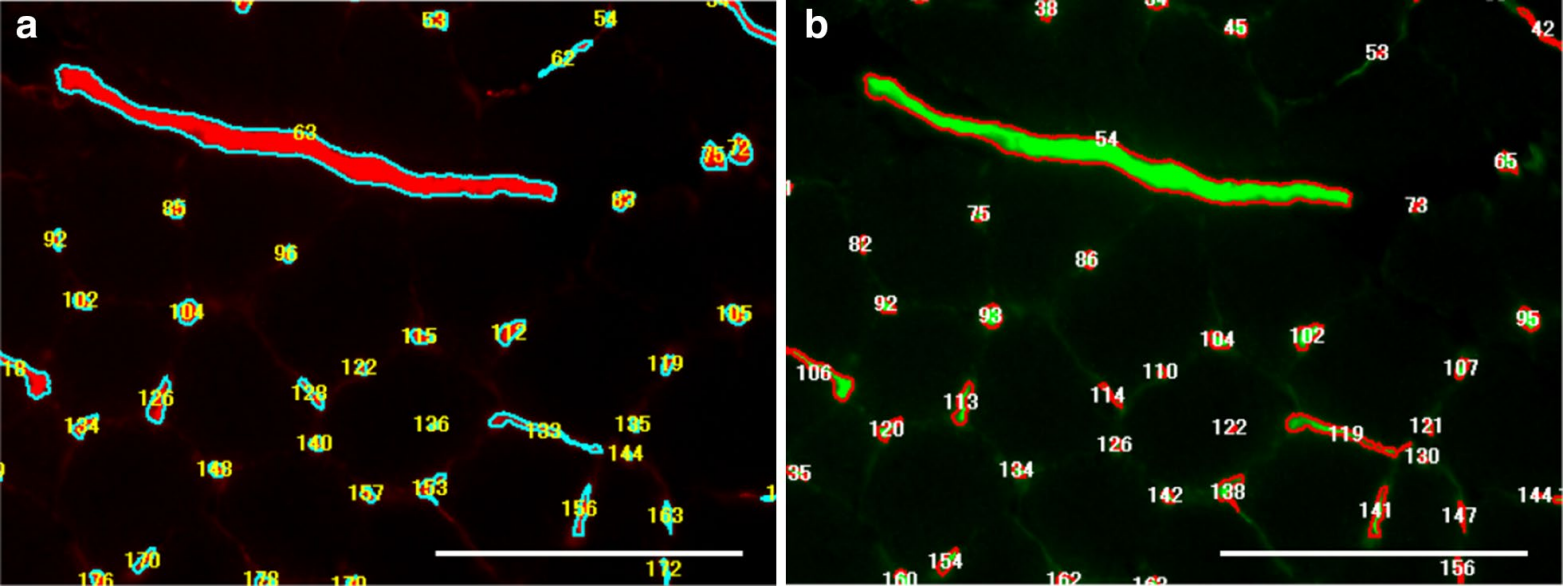
Semi-automatic counting of capillaries (an upper part of Figure 2a, b). Texas Red-positive (**a**) and FITC-positive (**b**) capillaries in the same field of a soleus muscle were automatically detected, numbered and counted after fine adjustment of threshold of the software. *Bars* 100 µm.

**Figure 2:**
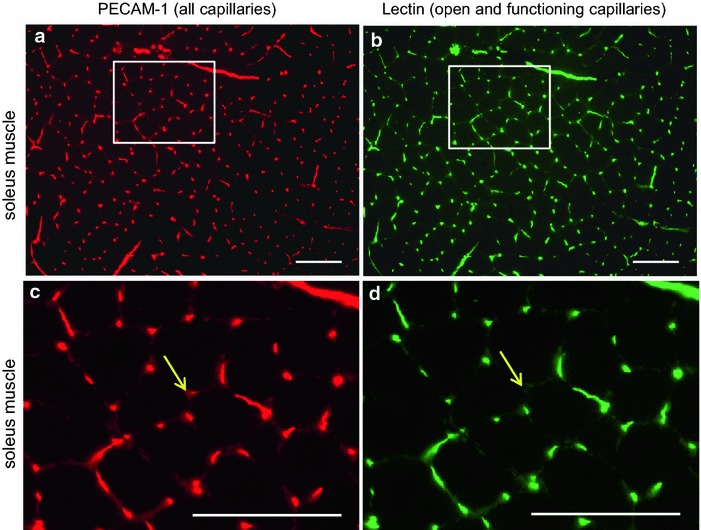
Micrographs of the soleus muscle. *Panels* **a** and **c** show staining for PECAM-1 (*red*) to detect all capillaries. *Panels* **b** and **d** show staining for FITC-lectin (*green*) to detect open and functioning capillaries. *Panels* **c** and **d** are higher magnification of rectangles in *panels* **a** and **b**, respectively. Most capillaries are FITC-lectin positive. No blood flow is observable in a capillary (*arrows*). *Bars* 100 µm.

**Figure 3:**
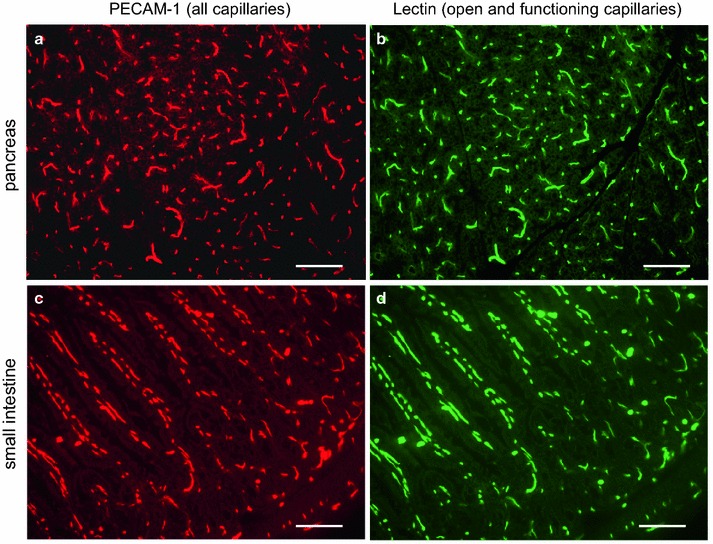
Micrographs of the pancreas and small intestine. *Panels* **a** and **c** show staining for PECAM-1. *Panels* **b** and **d** show staining for FITC-lectin. Most capillaries are FITC-lectin positive indicating blood flow. *Bars* 100 µm.

**Figure 4:**
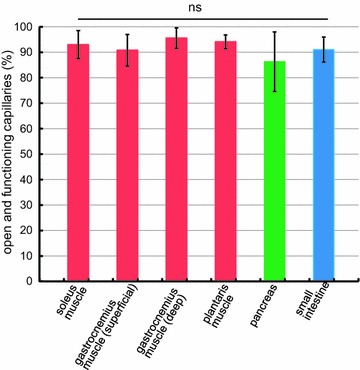
The proportions of open and functioning capillaries. The proportions are generally high and not significantly different among the leg muscles, pancreas and small intestine. *ns* not significant.

All specimens were clearly stained by immunostaining. The semi-automatic counting system in the present study needs much less time for measurements with minimal observer bias (Figure 1). The percentages of open and functioning capillaries were as follows: the soleus muscle, 93.0 ± 5.5% (Figure 2); the superficial zone of the gastrocnemius muscle, 90.8 ± 6.2%; the deep zone of the gastrocnemius muscle, 95.6 ± 4.0%; the plantaris muscle, 94.1 ± 2.7%; the pancreas, 86.3 ± 11.7%; and the small intestine, 91.1 ± 4.9% (n = 8, each) (Figure 3). These values were not significantly different from each other by the Kruskal–Wallis test (Figure 4).

## Discussion

In the present study, the proportion of open and functioning capillaries was semi-automatically measured using one section after double immunostaining. The results for skeletal muscles were generally in agreement with previous studies (Kayar and Banchero 1985; Kindig et al. 2002; Kano et al. 2005; Maeda et al. 2014). The proportions of open and functioning capillaries of skeletal muscles, pancreas and small intestine were high and not significantly different, in spite of their structural and functional differences. Our finding seems to support the notion that microvascular blood flow in various tissues and organs is mainly controlled by regulating the blood flow of each capillary, while the proportion of open and functioning capillaries remains relatively constant (Kindig et al. 2002; Maeda et al. 2014).

### Advantages of double-immunostaining of one section

In the case of skeletal muscle, most capillaries run along the long axis of muscle fibers. Thus, the profiles of the capillary bed are quite similar in serial cross sections. On the other hand, capillaries of the pancreas and small intestine run in various directions so the number, location and direction of capillaries can considerably differ from one section to another. Thus, the pancreas and small intestine were difficult to evaluate in terms of the proportion of open and functioning capillaries using paired sections. In the present study, however, *L. esculentum* lectin and PECAM-1 were sequentially stained in the same section. This method enabled us to examine various tissues and organs that have randomly oriented capillaries. In addition, the data of this method are probably more accurate and reliable than when measured by manual counting using paired sections stained for *L. esculentum* lectin and PECAM-1 separately in skeletal muscles (Maeda et al. 2014).

It is reported that anti-PECAM-1 antibody weakly stains lymphatic vessels (Ebata et al. 2001). Although there are lymphatic vessels (lacteals) in the villi of the small intestine, the proportion of open and functioning capillaries in the small intestine was almost comparable to those in the leg muscles and pancreas. This finding may indicate that lymphatic vessels do not interfere with measurements.

### Regulation of capillary blood flow

Capillary blood flow is influenced by blood flow velocity, diameter of blood vessels, and possibly systemic blood pressure. The velocity of muscle blood flow is considerably different from one capillary to another (Smaje et al. 1970), even at rest, and the velocity of blood flow drastically increases during muscle contraction (Honig et al. 1980; Kindig et al. 2002; Richardson et al. 2003). Furthermore, blood vessels dilate by axonal reflex and NO production by local heating (Minson et al. 2001). On the other hand, the arterioles are responsible for the vasoconstriction and a decrease of the velocity of blood flow (Lindbom et al. 1980).

Concerning the blood pressure, pentobarbital anesthesia (40 mg/kg) is reported to depress systemic blood pressure (91 mmHg, Field et al. 1993; 104 ± 2 mmHg, Kindig et al. 2002) compared with control rats (128 mmHg, Field et al. 1993). A decrease of the systemic blood pressure can lead to a reduction of the proportion of open and functioning capillaries; however, our results were high. Thus, it seems unlikely that pentobarbital anesthesia greatly changed the proportion of open and functioning capillaries.

The present study demonstrated that the proportions of open and functioning capillaries are high and similar in spite of structural and functional differences. This finding is in good agreement with the model proposed by Poole et al. (2013) and suggests that the microvascular systems of tissues and organs seem to share blood supply rather than a complete on–off capillary system. There is increasing evidence that most capillaries support blood flow in resting muscle and that precapillary sphincters are unlikely to exist except for the mesenteric microcirculation (Lindbom et al. 1980; Sakai and Hosoyamada 2013). Thus, the mechanism controlling the microvascular system needs to be clarified further.

## Conclusions

Considerable differences were reported in the blood flow in the leg muscle, pancreas and small intestine; however, the proportions of open and functioning capillaries were not significantly different in the present study. This finding indicates that the microvascular circulation in various tissues and organs is mainly controlled by changing of the blood flow in each capillary in spite of structural and functional differences, while the proportion of open and functioning capillaries was essentially unchanged.

## Methods

The present experimental procedures were approved by the Committee of Research Facilities for Laboratory Animal Science, Hiroshima University.

A total of eight Wistar male rats (254.6 ± 7.9 g, mean ± SD) aged 8 weeks old were used. The rats were anesthetized by an intraperitoneal injection of sodium pentobarbital (50 mg/kg) assisted by the inhalation of diethyl ether when necessary. The temperature of the right leg was monitored using a needle-type thermometer, TL3633 (25 mm long, diameter 1.3 mm; As One, Osaka, Japan), inserted into the hypodermis along the tibia. After tracheotomy and insertion of a breathing tube, mechanical ventilation was started and the left thorax was opened to expose the heart. A diluted solution of 200 µg/200 µl of FITC-labeled *L. esculentum* lectin (diluted 1:1 with saline, Vector, Burlingame, CA, USA) was injected into the bloodstream via the heart, mixed with blood and allowed to circulate in the whole body. This lectin is known to bind to vascular endothelium (Hashizume et al. 2000; Mazzetti et al. 2004) and 2–10 min was allowed for labeling blood vessels (Hashizume et al. 2000; Morikawa et al. 2002; Inai et al. 2004; Nakamura-Ishizu et al. 2008). Three minutes after the lectin injection, the heart was clamped using a hemostat to stop the blood flow. The right leg was maintained within 37.0 ± 0.8°C by cooling or warming during the period of lectin circulation. Recorded rectal temperature was within 37.0 ± 0.7°C. The soleus, gastrocnemius, plantaris muscles, pancreas and small intestine (presumably the jejunum) were removed. Muscles were transversally cut into three pieces and mid-belly portions were used. Muscles were aligned so that cross sections were cut and adhered to a piece of balsa wood (1 mm thick) that had been prefixed using glue at a right angle on a cork disk and coated with 6% tragacanth gum jelly just before use. The specimen and balsa wood on a cork disk were further supported with 6% tragacanth gum jelly and rapidly frozen in isopentane cooled with liquid nitrogen. The lumen of the small intestine was flushed with saline using a syringe and a tube. The small intestine and pancreas were half-embedded in 6% tragacanth gum jelly on cork disks and rapidly frozen. Cryosections (10 μm thick) were cut for immunostaining.

### Double-immunostaining

Cryosections were air-dried, rinsed three times for 5 min each to remove free FITC-lectin in 0.01 M phosphate-buffered saline (PBS, pH 7.2), fixed in cold acetone for 10 min and rinsed in PBS once for 5 min. Nonspecific binding sites were blocked for 30 min with a blocking solution containing 1% normal rabbit serum in PBS for FITC-lectin immunostaining. After blotting the blocking solution, the sections were incubated with a sheep anti-FITC antibody (1:500; Southern Biotech, Birmingham, AL, USA) for 2 h. Sections were rinsed with PBS three times for 5 min each and incubated for 1 h with rabbit biotinylated anti-sheep IgG antibody (1:100; Vector). Then, the sections were rinsed with PBS twice for 5 min each and incubated with FITC-labeled avidin (1:1,000; Vector) for 30 min. After two rinses with PBS for 5 min each, sections were treated with PBS containing 1% normal horse serum for 30 min for blocking of PECAM-1 immunostaining. After blotting, the sections were incubated with a mouse anti-PECAM-1 antibody (1:50; clone TLD-3A12, BD Bioscience, San Diego, CA, USA) for 2 h. Sections were rinsed with PBS three times for 5 min each and incubated for 1 h with horse biotinylated anti-mouse IgG antibody (1:100; Vector). Then, sections were rinsed with PBS twice for 5 min each and incubated with Texas Red-labeled avidin (1:50; Vector) for 30 min. After two rinses with PBS for 5 min each, sections were mounted with Vectashield (Vector). All procedures were conducted at room temperature. Sections skipping incubation with the primary antibody were all negative except for weak intrinsic fluorescence from FITC-labeled *L. esculentum* lectin. This weak fluorescence was unlikely to affect measurement because injected lectin was small in quantity. When 25 µg of FITC-labeled *L. esculentum* lectin was intravenously injected into a mouse, the liver was only lightly labeled (Robertson et al. 2015). In addition, fluorescence was much enhanced by immunostaining and the threshold of measurement was adjusted as described below.

### Calculation of the proportion of open and functioning capillaries

The center of each section of the soleus and plantaris muscles was photographed using a fluorescence microscope, Biorevo BZ-9000 (Keyence, Osaka, Japan), at a total magnification of 200. The gastrocnemius muscle was photographed at both superficial and deep zones separately because muscle fiber composition and capillary density differ between these zones. Each field was photographed twice: first with a filter set for FITC, and second with a filter set for Texas Red. Displaying these photographs on a computer display, one representative capillary that was clearly stained for both Texas Red and FITC was selected in each field. The number of pixels of the Texas Red-positive area of the selected capillary was measured using the software BZ II (Keyence) installed in the microscope system. Next, the number of pixels of the FITC-positive area of the selected capillary was measured. Then, the threshold of the software was adjusted so that the number of pixels of the FITC-positive area of the selected capillary approached the number of pixels of the Texas Red-positive area of the capillary as closely as possible. Using the software BZ II, Texas Red-positive areas (all capillaries) and FITC-positive areas (open and functioning capillaries), regardless of longitudinal or cross sections of capillaries, were automatically detected, numbered and counted (Figure 1). At least 170 (range 170–554) capillaries were examined in each specimen. To calculate the proportion of open and functioning capillaries, the number of FITC-positive areas was divided by the number of Texas Red-positive areas and multiplied by 100.

### Statistical analysis

All data are presented as mean ± SD. Data distribution was assessed via the Shapiro–Wilk test for normality. To test whether the samples were from populations with equal variances, Bartlett’s test was used. Because normality and dispersion were not equal, the Kruskal–Wallis test was used. A statistical significance level of p < 0.05 was accepted.
